# Progress of Binder Structures in Silicon-Based Anodes for Advanced Lithium-Ion Batteries: A Mini Review

**DOI:** 10.3389/fchem.2021.712225

**Published:** 2021-10-12

**Authors:** Wenqiang Zhu, Junjian Zhou, Shuang Xiang, Xueting Bian, Jiang Yin, Jianhong Jiang, Lishan Yang

**Affiliations:** ^1^ Key Laboratory of Chemical Biology and Traditional Chinese Medicine Research (Ministry of Education of China), National and Local Joint Engineering Laboratory for New Petrochemical Materials and Fine Utilization of Resources, Key Laboratory of the Assembly and Application of Organic Functional Molecules of Hunan Province, Hunan Normal University, Changsha, China; ^2^ Hunan Engineering Research Center for Water Treatment Process and Equipment, China Machinery International Engineering Design and Research Institute Co., Ltd., Changsha, China

**Keywords:** binder, Si-based anode, lithium-ion batteries, structure, cross-linked

## Abstract

Silicon (Si) has been counted as the most promising anode material for next-generation lithium-ion batteries, owing to its high theoretical specific capacity, safety, and high natural abundance. However, the commercial application of silicon anodes is hindered by its huge volume expansions, poor conductivity, and low coulombic efficiency. For the anode manufacture, binders play an important role of binding silicon materials, current collectors, and conductive agents, and the binder structure can significantly affect the mechanical durability, adhesion, ionic/electronic conductivities, and solid electrolyte interface (SEI) stability of the silicon anodes. Moreover, many cross-linked binders are effective in alleviating the volume expansions of silicon nanosized even microsized anodic materials along with maintaining the anode integrity and stable electrochemical performances. This mini review comprehensively summarizes various binders based on their structures, including the linear, branched, three-dimensional (3D) cross-linked, conductive polymer, and other hybrid binders. The mechanisms how various binder structures influence the performances of the silicon anodes, the limitations, and prospects of different hybrid binders are also discussed. This mini review can help in designing hybrid polymer binders and facilitating the practical application of silicon-based anodes with high electrochemical activity and long-term stability.

## Introduction

With the increasing energy-storage demands of electric vehicles and portable electronic devices, conventional commercial graphite anodes are becoming unsatisfied with the requirements of high energy density batteries (Li et al., 2018; Zeng et al., 2019). Next-generation lithium-ion batteries (LIBs) with silicon (Si) anodes are promising owing to their high theoretical specific capacity (∼4,200 mA h g^−1^) along with nontoxicity and abundance in nature (Chae et al., 2020; Chen et al., 2021; Ge et al., 2021; Zhao et al., 2021). Nowadays, several issues such as huge volume expansions, low conductivity, and unstable SEI film hinder the real application of Si anodes (Son et al., 2015; Franco Gonzalez et al., 2017; Huang et al., 2021; Yang et al., 2021). To address these challenges, researchers mainly concentrate on the design of Si nanomaterials and the optimization of electrolytes but pay fewer attention to the binders (Lee et al., 2021). It seems that the binders account for a small proportion in anode composition without direct contribution to the capacity, but actually, binders play an important role in maintaining the inert electrical contact and the SEI stability of anodes, which will affect the anodes in porosity, wettability, electronic/ionic conductivities, polarization, etc.

Numerous single-component binders with linear structures have been reported, such as polyvinylidene fluoride (PVDF) (Li et al., 2008; Huang et al., 2021), polyacrylic acid (PAA) (Magasinski et al., 2010; Parikh et al., 2019; Hu et al., 2021), carboxymethyl cellulose (CMC) (Drofenik et al., 2003; Wu and Li, 2020), sodium alginate (SA) (Kovalenko et al., 2011; Cai et al., 2019), polymerized styrene butadiene rubber (SBR) (Li et al., 2007), polyvinyl alcohol (PVA) (Park et al., 2011; Mandal et al., 2021), chitosan (CS) (Yue et al., 2014; Rajeev et al., 2020), polyacrylonitrile (PAN) (Luo et al., 2016), polyimide (PI) (Kim et al., 2013; Lee et al., 2018), gum arabic (GA) (Ling et al., 2015; He et al., 2021; Zhong et al., 2021), and guar gum (GG) (Liu et al., 2015; Zhao et al., 2021). Properly designed binders with hybrid structures, such as branched binders and cross-linked binders, show broader feasibility for improving the electrochemical performance of Si-based LIBs(Preman et al., 2020). The key factor why the binders could affect Si anodes is their structural characteristics (Zhao et al., 2021). For example, polar groups within binders can form hydrogen, covalent, or ionic bonds with surficial silanol groups or SiO_x_, which can maintain the adhesion among the anodic components (including Si particles, conductive agents, and current collectors), enhance the mechanical integrity of the electrode, and finally support the Si anodes with good electrical conductivity and cycling stability.

Nowadays, an increasing number of binders, with features such as ultrahigh elasticity, superior chemical stability, self-healing, and good conductivity, have been developed for advanced Si-based anodes. Herein, this mini review has concisely summarized recent advances on various binders for Si-based anodes according to their structures (e.g., linear, branched, conductive polymer, 3D cross-linked, and other hybrid binders). Special attention has been paid to how those hybrid structures and functional groups influence the performance of Si-based anodes. In addition, the design directions of novel hybrid binders and new research methods of electrodes have been discussed in this mini review.

## Binders in Si-Based Anodes

**FIGURE 1:**
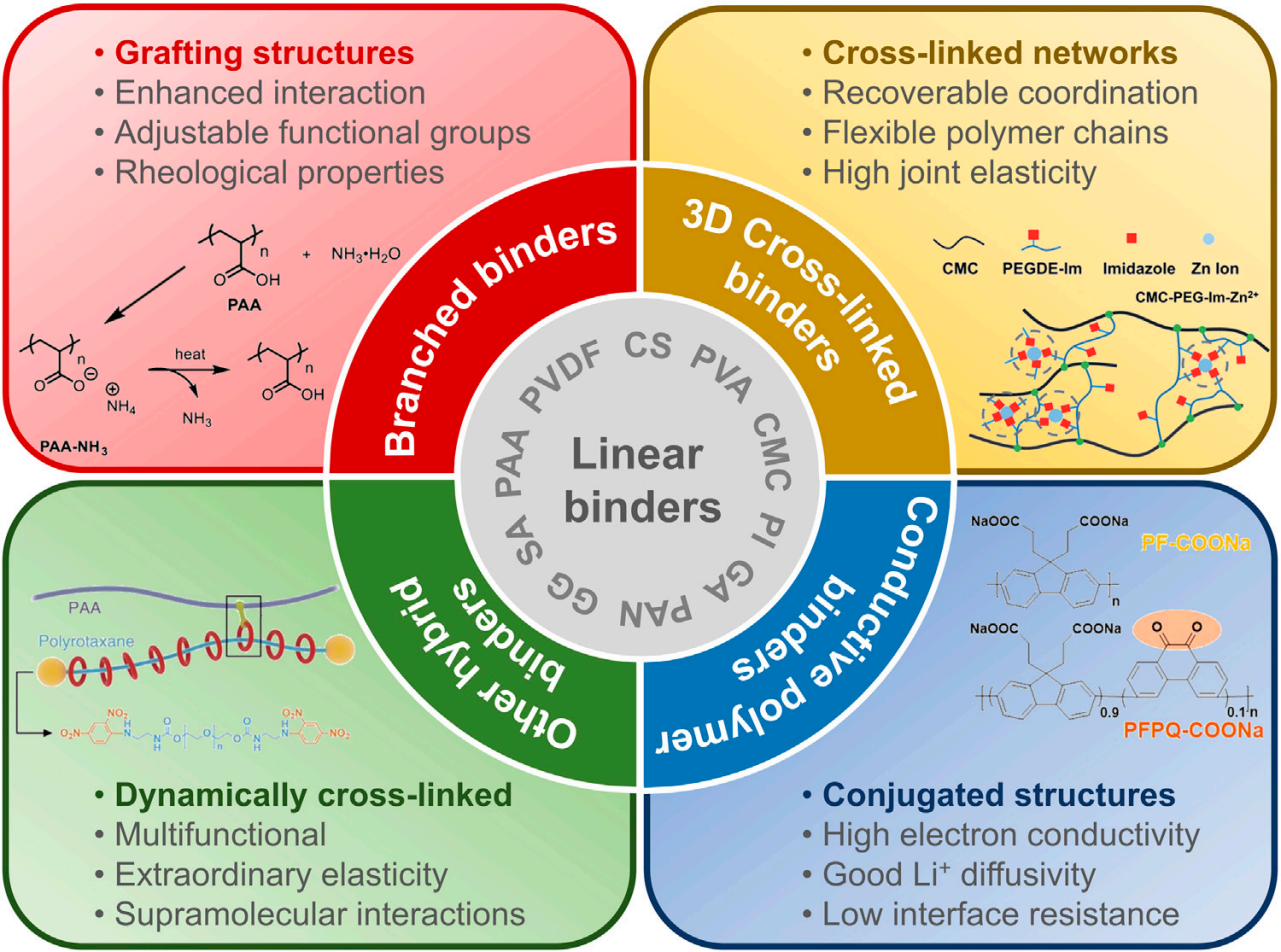
An overview of various binders for Si-based anodes based on polymer structures. Basically, binders are divided into linear and hybrid structures (e.g., branched, 3D cross-linked, conjugated, dynamically cross-linked, and others). The inserted graphics are adapted with permission from Choi et al., (2017); Zhao et al., (2018); Shi et al., (2020); and Kim et al., (2021).

**FIGURE 2:**
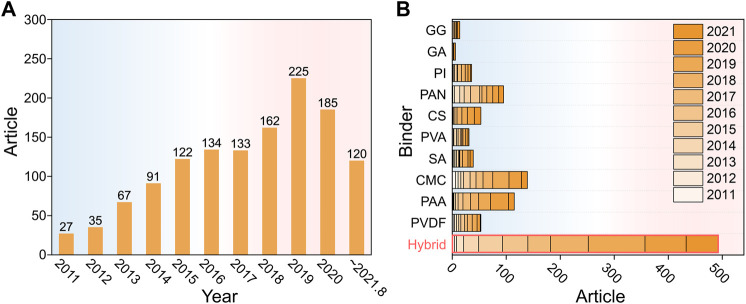
The number of publications on binders for Si-based anodes from year 2011 to August 2021. **(A)** The overall numbers for binders of Si-based anodes. **(B)** Subdivided data by 10 typical linear binders and hybrid binders (mentioned in Figure 1). Data were collected from Web of Science in August 2021.

The structures of binders would affect the Si-based anodes through multiple interactions, such as chemical bonding, mechanical interlocking, conjugate conductivity, van der Waals forces, and electrostatic forces, which are mainly related with their backbone structures and functional groups of the binders (Chae et al., 2020; Zhao et al., 2021). Based on the polymer structures, binders for Si-based anodes can be simply divided into five categories, namely, linear, branched, 3D cross-linked, conductive polymer, and other hybrid binders (Figure 1). The detailed discussion will be carried out in the following sections. In Figure 2A, we searched the published articles on Si-anode binders in the past 11 years (from January 2011 to August 2021). The data are obtained from Web of Science, by using the keywords: silicon anode, binder, and the name of a specific binder. Due to the inability to search the full text (especially the experimental part), the literatures listed in Figure 2B are studying the binders instead of just using a certain binder, which makes the data of Figure 2B much less than the overview of Figure 2A. Obviously, developing hybrid binders is the most promising choice for the application of Si anodes.

### Single-Component Binders With Linear Structures

Generally, natural and synthetic single-component binders have linear structures. By now, many single-component binders have been exploited and applied to Si-based anodes for advanced LIBs. Meanwhile, these binders have polar functional groups that can form hydrogen, covalent, or ionic bonds with silanol or SiO_x_ on the surface of Si, suppressing the volume expansions of Si during charging/discharging for a good cyclic life and excellent rate performances and, in some cases, providing electronic and ionic conductivity. The properties, active functional groups, and structural formulas of typical binders are listed in Tables 1 and 2.

**TABLE 1:** The structures and key properties of various binders for Si-based anodes.

Binder	Feature	Structure	Active site	Structural formula	Ref.
PVDF	Poor mechanical property, poor elasticity	Linear	Fluorine atom	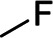	Li et al. (2008); Huang et al. (2021)
PAA	High viscosity, high mechanical properties	Linear	Carboxyl group	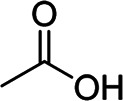	Magasinski et al. (2010); Parikh et al. (2019)
CMC	High rigidity, high brittleness	Linear	Hydroxyl group, ester bond	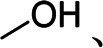	Lestriez et al. (2007); Wu and Li (2020)
				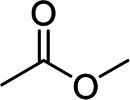	
SA	High viscosity	Linear	Hydroxyl group, ester bond	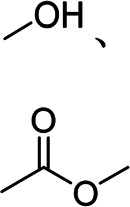	Kovalenko et al. (2011)
PVA	High adhesion	Linear	Hydroxyl group	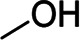	Park et al. (2011)
CS	Water-soluble, strong hydrogen bonding	Linear	Hydroxyl group, amino group	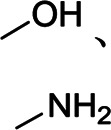	Yue et al. (2014)
PAN	High adhesion, high electronic/ionic conductivity	Linear	Nitrile group	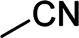	Luo et al. (2016)
PI	High mechanical property	Linear	Carbonyl group	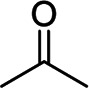	Kim et al. (2013)
GA	High mechanical property	Linear	Hydroxyl group	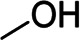	Ling et al. (2015)
GG	High viscosity, good ion conductivity	Linear	Hydroxyl group	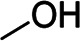	Liu et al. (2015)
Alg-C	Extraordinary wetness-resistant adhesion	Branched	Hydroxyl group, peptide bond	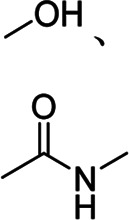	Ryou et al. (2013)
CS/rubber	Elasticity, high adhesion	Branched	Hydroxyl group, amino group	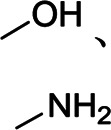	Lee et al. (2018)
PAA-NH_3_	High viscosity, strong shear-thinning effects	Branched	Ammonium carboxylate group	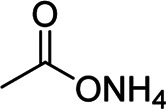	Shi et al. (2020)
*β*-CDp	Hyperbranched network structure, multidimensional hydrogen-bonding interactions	Branched	Hydroxyl group	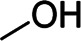	Jeong et al. (2014)
c-Alg-g-PAAm	Dual cross-linked network	Branched	Peptide bond, calcium ion	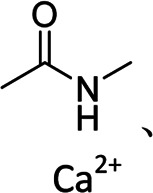 , Ca^2+^	Gendensuren and Oh (2018)
c-PAA-CMC	Three-dimensionally interconnected network	3D cross-linked	Hydroxyl group, ester bond	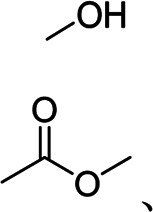	Koo et al. (2012)
PAA-PVA	Deformable polymer network, strong adhesion	3D cross-linked	Hydroxyl group, ester bond	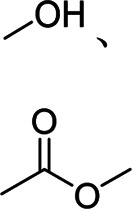	Song et al. (2014)
b-Li_0.5_PAA@SA	Bicontinuous composite network	3D cross-linked	Hydroxyl group, ester bond	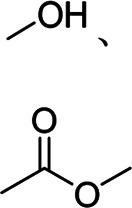	Hu et al. (2021)
cxPAA-PEI(x)	Superior mechanical properties, good adhesion	3D cross-linked	Peptide bond	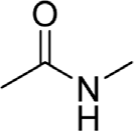	Chuang et al. (2021)
CMC-co-SN	Excellent binding interaction	3D cross-linked	Thiourea group	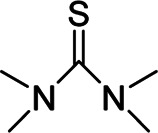	Ren et al. (2021)
SBR/CMC-PEG-Im-Zn^2+^	Tight interparticle contacts, high elasticity	3D cross-linked	Ester bond zinc ion	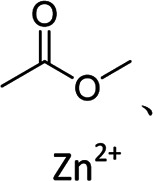	Kim et al. (2021)
CS-grafted-PANI	Electrically conductive, mechanically robust	Conjugated	Hydroxyl group, imine group	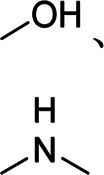	Rajeev et al. (2020)
PR-PAA	Extraordinary elasticity	Dynamic cross-linked	Hydroxyl group	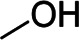	Choi et al. (2017)
SA/Ca^2+^	High mechanical property	3D non–cross-linked	Hydroxyl group, ester bond, calcium ion	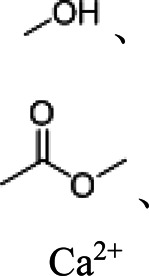	Liu et al. (2014)

**TABLE 2:** The electrochemical performances for Si-based anodes with various binders.

Binder	Capacity (mAhg^−1^)/current/(mAg^−1^)	Binder content (wt%)	Capacity retention	Initial Coulombic efficiency (%)	Ref.
PVDF	2000/150	8	>600 mAh g^−1^ after 50 cycles at 150 mA g^−1^	—	Li et al. (2008)
PVDF	∼3000/210	15	1900.6 mAh g^−1^ after 200 cycles at 0.2C	75.32	Huang et al. (2021)
PAA	>3300/210	∼15	94% after 20 cycles at C/20	—	Magasinski et al. (2010)
PAA	3650/210	25	80% after 100 cycles at C/10	84.6	Parikh et al. (2019)
CMC	2656/210	15	2,373 mAh g^−1^ after 55 cycles at 0.2C	—	Wu and Li (2020)
CMC	∼4000/150	8	∼300 mAh g^−1^ after 15 cycles at 150 mA g^−1^	—	Lestriez et al. (2007)
SA	∼2000/4200	15	>1700 mAh g^−1^ after 100 cycles at 4,200 mA g^−1^	—	Kovalenko et al. (2011)
PVA	∼1300/—	10	∼600 mAh g^−1^ after 50 cycles at C/2	—	Park et al. (2011)
CS	4270/200	8	950 mAh g^−1^ after 50 cycles at 500 mA g^−1^	89	Yue et al. (2014)
PAN	4147.8/100	10	1,639.6 mAh g^−1^ after 50 cycles at 100 mA g^−1^	86.2	Luo et al. (2016)
PI	2077/200	10	75% after 20 cycles at 200 mA g^−1^	81	Kim et al. (2013)
GA	4056/—	25	2,708 mAh g^−1^ after 100 cycles at C/10	88.11	Ling et al. (2015)
GG	3364/1000	15	2,222 mAh g^−1^ after 100 cycles at 2,100 mA g^−1^	88.3	Liu et al. (2015)
Alg-C	∼1700/420	20	∼100% after 400 cycles at 2,100 mA g^−1^	—	Ryou et al. (2013)
CS/rubber	∼3600/400	15	1,350 mAh g^−1^ after 1,600 cycles at 8,000 mA g^−1^	81.4	Lee et al. (2018)
PAA-NH_3_	711/210	10	∼60.2% after 100 cycles at C/3	96.5	Shi et al. (2020)
*β*-CDp	2142/420	20	1,471 mAh g^−1^ after 200 cycles at 4,200 mA g^−1^	85.9	Jeong et al. (2014)
c-Alg-g-PAAm	∼1160/—	15	836 mAh g^−1^ after 100 cycles	—	Gendensuren and Oh (2018)
PVDF-b-PTFE	1395/420	5	∼1,000 mAh g^−1^ after 200 cycles at 1C	82.7	Wang et al. (2020)
c-PAA-CMC	∼2900/175	20	∼2,100 mAh g^−1^ after 100 cycles at 300 mA g^−1^	88	Koo et al. (2012)
PAA-PVA	3616/400	20	2,283 mAh g^−1^ after 100 cycles at 400 mA g^−1^	86	Song et al. (2014)
b-Li_0.5_PAA@SA	2762/210	20	1,584 mAh g^−1^ after 150 cycles at 0.1C	∼86	Hu et al. (2021)
ReDNA/Alg	∼2400/350	20	∼2000 mAh g^−1^ after 160 cycles at 1750 mA g^−1^	84.6	Kim et al. (2018)
cxPAA-PEI(x)	∼3500/210	20	2,606 mAh g^−1^ after 100 cycles at 0.2C	86.68	Chuang et al. (2021)
CMC-co-SN	∼3000/210	15	1,083 mAh g^−1^ after 300 cycles at 840 mA g^−1^	83.2	Ren et al. (2021)
S-PA	2736/—	15	2,295 mAh g^−1^ after 200 cycles at 2,100 mA g^−1^	∼87	Zhang et al. (2021)
SBR/CMC-PEG-Im-Zn^2+^	∼540/65	10	100.8% after 200 cycles at 325 mA g^−1^	∼97	Kim et al. (2021)
PAA-FREP	∼2800/300	15	∼2,400 mAh g^−1^ after 320 cycles at 1,000 mA g^−1^	85.7	Liu et al. (2020)
PAA-UPy	4194/210	20	2,638 mAh g^−1^ after 110 cycles at 840 mA g^−1^	86.4	Zhang et al. (2018)
CS-grafted-PANI	4417/—	20	1,091 mAh g^−1^ after 200 cycles at 1C	72.4	Rajeev et al. (2020)
c-PEO-PEDOT:PSS/PEI	2440/1000	20	2027 mAh g^−1^ after 500 cycles at 1,000 mA g^−1^	82.0	Zeng et al. (2018)
PFPQ-COONa	1925/210	∼33.3	901 mAh g^−1^ after 400 cycles at 2,100 mA g^−1^	∼71	Zhao et al. (2018)
PPyMAA	3928.8/420	10	2,200 mAh g^−1^ after 180 cycles at 420 mA g^−1^	82.08	Zheng et al. (2019)
PR-PAA	2971/100	10	91% after 150 cycles at 600 mA g^−1^	97.37	Choi et al. (2017)
Alginate hydrogel	∼2800/100	29	82.3% after 120 cycles at 420 mA g^−1^	—	Liu et al. (2014)

1C = 4,200 mA g^−1^.

#### Natural Binders

The CMC binder is one of the most representative linear binders for Si-based anodes, which has good stability, low price, safety, and environmental friendliness. The key factor that the CMC binder can maintain long-cycling stability of anodes is that the CMC binder is a sticky linear polysaccharide molecule with hydroxyl and carboxyl groups. Then, CMC can form hydrogen and covalent bonds with surficial silanol/SiO_x_ on Si particles and inhibit the decomposition of the electrolyte on the silicon interface (Yilmaz et al., 2012; Jung et al., 2013). In 2003, the CMC binder was firstly applied to graphite anodes for LIBs (Drofenik et al., 2003). Along with 2 wt% CMC binder, the anodic properties exhibit reversible specific capacity (>300 mA h g^−1^) during the first 10 cycles, and the kinetics of the Li^+^ insertion/desertion process seem not to be affected, inspiring the CMC binder for Si-based anodes. Courtel et al. demonstrated that the electrochemical performances were significantly affected by the degree of CMC substitution of the molecular structures and the length of the polymeric chain (Courtel et al., 2011). Moreover, the extension of a CMC linear molecular chain in the solution is critical to help to form a network structure during electrode processing (Lestriez et al., 2007). Recently, Wu et al. found that the pure CMC binder is uniformly distributed in the drying/thickness direction of the electrode sheet, but SBR/CMC tends to migrate and finally accumulate on the top surface of the electrode (Wu and Li, 2020).

Compared with nonelastic CMC, SA possesses higher viscosity owing to more uniformly distributed carboxyl groups, which is widely utilized in Si anodes. Kovalenko et al. first employed an SA binder and got a stable Si anode during 1,300 cycles at 1.2 A g^−1^, owing to high tensile strength, uniformly distributed carboxyl groups, and the stable SEI (Kovalenko et al., 2011). Chitosan is one of the most abundant natural polysaccharides as same as CMC in nature. Zhang et al. treated the slightly water-soluble chitosan through the carboxymethylation reaction to obtain water-soluble carboxymethyl chitosan and exploited carboxymethyl chitosan as a good binder for Si-based anodes (Yue et al., 2014). FTIR and XPS testing demonstrated the formation of stable hydrogen bonds between polar groups (−OH, −COOH, and −NH_2_) of carboxymethyl chitosan and Si. The first discharging capacity was ∼4,270 mA h g^−1^ with high initial coulombic efficiency (85%) but poor stability.

#### Synthetic Binders

Numerous synthetic binders have been exploited with the commercialization of LIBs, where PVDF was one of the first commercial binders. Li et al. prepared the anodes with PVDF binders, which had a first discharging capacity of 2000 mA h g^−1^ (Li et al., 2008). For the formation of a stable SEI layer, Huang et al. employed a supramolecular layer of nickel and ion-trimesic acid as a functional buffer layer on the Si particle surface to protect Si particles from direct contact with the electrolyte or PVDF chains, leading to much improved initial coulombic efficiency and high-rate capability. However, the weak van der Waals force between PVDF and Si-based active materials could not withstand the volume expansions of silicon in long cycles (Huang et al., 2021). To avoid the use of toxic organic electrolytes, the application of PVDF in Si-based anodes gradually disappeared. PVA was first employed as a Si-based anodic binder of lithium-ion LIBs by Park et al. With numerous hydroxyl groups, PVA could form strong hydrogen bonds with both Si and the current collector to enhance the adhesion strength of electrodes (Park et al., 2011). However, hydrogen bonds are not strong enough to withstand the repeated volume expansions of Si, causing poor cyclic stability in PVA cases. Compared with PVDF and PVA, PAA is a nontoxic water-soluble binder with both hydrogen bonds and covalent bonds with the Si surface. Magasinski et al. found that PAA binders have a high concentration of carboxyl groups and strong mechanical properties (Magasinski et al., 2010). Compared with SA, PAA has strong water absorption, affecting the performances of the batteries. Besides, a few of single-component binders with nonlinear structures are reported, such as the small-molecule networked tannic acid binder (Sarang et al., 2020) and three-dimensional polyurethane binder (Wu et al., 2021).

In general, most single-component binders for Si-based anodes of LIBs are linear, which means there are few interaction sites between the binders and Si materials; thus, there is lack of strength to withstand the volume expansions of Si crystals. Besides, the polar groups of single-component binders can form hydrogen or covalent bonds with Si, which may be reversible with self-healing effects but do not contribute to long cycling stability. Compared with hydrogen bonds, covalent bonds have stronger mechanical properties, but they are irreversible once broken, which could lead to cracking and pulverization, poor electrical contact, and unstable growth of SEI of the anodes. In addition, most single-component binders have no contribution to the conductivity of the anodes. Therefore, reconstructing single-component binders into hybrid binders is expected to solve these issues of Si-based anodes.

### Hybrid Binders

In this work, we classify the hybrid binders into branched, 3D cross-linked, conductive polymer, and other hybrid binders based on their structures (Cao et al., 2019; Chen et al., 2020). Among them, 3D cross-linked hybrid binders can provide more connection sites and more void space between binders and Si particles to gain higher mechanical properties and proper Li^+^ conduction pathways. Although 3D cross-linked hybrid binders seem to be the most promising binders for Si-based anodes, the operations of grafting, cross-linking, and dynamically cross-linking will be interactively used in the design of new hybrid binders. Next, we will introduce them one by one as shown in Figure 1.

Inspired by the high adhesion of mussels in water, Ryou et al. (2013) prepared hybrid binders of dopamine hydrochloride–grafted PAA (PAA-C) and sodium alginate (Alg-C). Due to the fact that linear PAA and Alg became grafted PAA-C and Alg-C, respectively, there were more hydrogen bonds to improve mechanical properties and the contact area providing more active materials for lithiation/delithiation to increase reversible capacity between binders and Si particles. Compared with those of the pure PAA and Alg binders, the cycling stability and reversible capacity of the electrodes with PAA-C and Alg-C hybrid binders were improved. Based on the fact that binders with high viscosity such as PAA and SA are generally with low mechanical properties, Lee et al. utilized irregular branched hybrid binders composed of highly elastic natural rubber and highly viscous chitosan in Si-based anodes for LIBs (Lee et al., 2018). The existence of hydrogen bonds that are between Si particles and chitosan rich in amino and hydroxyl groups along with the elasticity of natural rubber can maintain the stability of the electrode structures. To enhance adhesion strength and flexibility, He et al. synthesized gum arabic–grafted poly (acrylic acid) (GA-g-PAA) binders for Si anodes in LIBs *via* a free radical reaction. Compared with the Si electrode of a pure PAA binder, the GA-g-PAA electrode exhibited better cyclic stability, higher Coulombic efficiency, and superior rate properties (He et al., 2021). Similarly, Shi et al. developed poly grafted ammonia (acrylic acid) (PAA-NH_3_) binders to improve cycling performances with favorable rheological properties of slurries in Si anodes for LIBs due to the fact that the resulting ammonium carboxylate groups may cleave during the drying process to restore the neutralized PAA (PAA-NH_3_) binders to their pristine states (Shi et al., 2020).

In order to further increase the strength of the hydrogen bonds between binders and Si particles, Jeong et al. first introduced the hyperbranched *β*-cyclodextrin polymer (*β*-CDp) as a new binder in Si-based anodes for LIBs. From a practical viewpoint, *β*-CD could be easily synthesized from ordinary starch by established enzymatic processes. Meanwhile, the hyperbranched network structure of *β*-CDp enables the formation of multidimensional hydrogen bonds between binders and Si particles, thus causing excellent cycling stability. With the existence of hydrogen bonds, the *β*-CDp–based electrode exhibited a self-healing effect, leading to long-term cycling stability (Jeong et al., 2014). Oh et al. synthesized a dual cross-linked alginate with polyacrylamide (c-Alg-g-PAAm) branched binders in Si anodes for LIBs. The polyacrylamide provided strong adhesion in the electrode with resistance to the penetration of the organic electrolyte. And, ionic and covalent cross-linkings in the binder could maintain their intrinsic good binding properties and additionally enhance lithium-ion diffusion, thus leading to excellent cyclability (Gendensuren and Oh, 2018) Similarly, Wang et al. developed a tough block copolymer PVDF-b-teflon (PTFE) binder that could coalesce pulverized Si and thus enhanced the stability of Si anodes (Wang et al., 2020).

Weak hydrogen bonds between binders and Si particles cannot withstand the volume expansions during long cycles. To address this issue, researchers exploit 3D cross-linked hybrid binders to form stronger covalent bonds for higher mechanical properties and more contact area between binders and active materials. In addition, 3D cross-linked structures reduce the contact between the electrodes and the electrolyte for a stable SEI film and provide void space for volume expansions and deinsertion/insertion of Li ions. In other words, cross-linked binders *via* chemical bonds can effectively fix the electrode particles, inhibit the volume expansion of Si particles, and stabilize the solid electrolyte interface, thus enabling good cycling stability of silicon anode–based batteries (Chen et al., 2020). For example, Koo et al. utilized a hybrid binder c-PAA-CMC heated at 150°C for 2 h under vacuum to prepare Si-based electrodes (Koo et al., 2012). After esterification, covalent ester bonds and hydrogen bonds were formed between the carboxyl groups of PAA and the hydroxyl groups of CMC along with the silanol groups on the surface of Si, thus forming the 3D cross-linked structure. Especially, the anhydride could be generated in PAA chains by the dimerization of carboxlic acid, leading to stability of electrode structures. Therefore, enhanced interaction between the c-PAA-CMC binder and Si particles further inhibited irreversible movement of Si particles and maintained good electrical contact during long cycles. Besides, the elimination of hydroxyl groups on the surface of Si particles alleviated capacity loss by the electrochemical reaction with lithium, and the 3D cross-linked structure preserved void space for the volume expansions of Si and deinsertion/insertion of Li ions, which contributed to the high coulombic efficiency and long-term stability. Inspired by the recovery of resin to its original state after losing the solvent from its 3D flexible network, Song et al. prepared a gel polymer binder for Si anode materials through an *in situ* thermal cross-linking of water soluble PAA and PVA at 100°C for 5 h and then at 150°C for another 1 h under vacuum (Song et al., 2014). Carboxyl groups of PAA and the hydroxyl groups of PVA along with the silanol groups on the surface of Si formed covalent ester bonds and hydrogen bonds. In addition, resin could change with the volume of Si. Therefore, PAA-PVA hybrid binders have the similar high mechanical behavior and superior electrochemical performances with c-PAA-CMC binders. Pretreatment of binder, current collector, and active material before cross-linking is confirmed to be a good way to improve the performance of silicon anodes (Huang et al., 2018; Kim et al., 2018; Jung et al., 2019; Cao et al., 2021; Chuang et al., 2021; Hu et al., 2021; Kim et al., 2021; Ren et al., 2021; Zhang et al., 2021). For example, lithiated polyacrylate acid (Li_x_PAA) (Hu et al., 2021), treatment of silicon powder with piranha solution (Jung et al., 2019), partially neutralized PAA (Huang et al., 2018), and renatured DNA (Kim et al., 2018) can get further stretched structure and enhanced contact between electrode materials and the Cu current collector to achieve advanced Si anodes. In order to get LIBs with high energy density and good safety, Liu et al. designed a three-dimensional flame-retardant binder (PAA-FREP) by cross-linking polyacrylic acid (PAA) with a flame-retardant epoxy resin (FREP) containing phosphorus and nitrogen elements, which exhibited excellent mechanical, electrochemical, and safety performances (Liu et al., 2020).

For superior self-healing, self-healing binders are reported in recent years (Xu et al., 2018; Zhang et al., 2018; Chen et al., 2021). Zhang et al. prepared a quadruple hydrogen-bonded supramolecular binder PAA-UPy through covalently integrating a small amount of ureido-pyrimidinone (UPy) moieties with PAA (Zhang et al., 2018). Due to its quadruple hydrogen-bonding dynamic interaction, the PAA-UPy binder exhibited an excellent self-healing ability. Xu et al. designed a PAA-P(HEA-co-DMA) binder by an *in situ* thermal condensation of PAA with hydroxyethyl acrylate and dopamine methacrylate. Due to the existence of carboxyl, catechol groups, covalent, and noncovalent cross-linking, the PAA-P(HEA-co-DMA) binder not only showed strong interactions with active materials and adhesion force but also provided good mechanical properties and high flexibility (Xu et al., 2018).

The conductive additives are generally added to Si anodes for good conductivity, but they will disconnect with Si particles during the continuous volume change and contribute no capacity to the cell (Zhao et al., 2021). Conductive polymer binders are expected to address this issue (Zhao et al., 2015; Zhao et al., 2015; Wang et al., 2018; Zeng et al., 2018; Zhao et al., 2018; Zheng et al., 2019; Rajeev et al., 2020). Kim et al. synthesized electrically conductive and mechanically robust polymer binder chitosan-grafted polyaniline copolymers (CS-g-PANI) for Si anodes, showing well-balanced electrical conductivity, mechanical properties, and the best cell performances when the CS-g-PANI binder was composed of 50% CS and 50% PANI (Rajeev et al., 2020). Zeng et al. designed a conductive polymer binder c-PEO-PEDOT:PSS/PEI by assembling ion-conductive PEO and PEI polymers onto high electron-conductive PEDOT:PSS chains by chemical cross-linking, chemical reduction, and electrostatic self-assembly. This polymer binder showed lithium-ion diffusivity and electron conductivity that were 14 and 90 times higher than those of the CMC (with acetylene black) binder, respectively (Zeng et al., 2018).

### Other Hybrid Binders

In order to realize the multifunctions of binders, some recent hybrid binders have comprehensively introduced polymer operations such as grafting, cross-linking, and dynamically cross-linking. For example, dynamically cross-linked binders (e.g., PR-PAA) with high elasticity and tolerance combine the best of covalent and noncovalent cross-linking in the form of mechanical bonds, which preserved the dynamic nature of the polymer networks (Kwon et al., 2018). Choi et al. prepared a PR-PAA binder with unique structures, which was inspired by the principle of pulley (Choi et al., 2017). The ring *α*-cyclodextrin could form covalent bonds with the chained PAA that can slide freely on the polyrotaxane molecular chain. Although with a low binder content (10 wt%), the PR-PAA binders also had high viscosity and elasticity to withstand the volume expansions of Si particles, maintaining the stability of electrodes. Furthermore, Choi et al. developed a pyrene–poly (acrylic acid)–polyrotaxane supramolecular binder based on the mechanism of ring pulley, which helps to get a high-performance silicon anode (Cho et al., 2019). Because SA can adsorb metal ions to form a hydrogel in aqueous solution, Liu et al. first employed an SA hydrogel as a Si-based anode binder through the cross-linked effect of SA with Ca^2+^ for LIBs (Liu et al., 2014). The introduction of Ca^2+^ increased the electrostatic interaction inside the SA hydrogel and formed networks to accommodate void space for the volume expansions of Si particles, where the capacity of this anode was 1822 mAh g^−1^ even at 420 mA g^−1^ after 120 cycles.

Multifunctional binders can be synthesized with various advantages, including strong adhesion, high elasticity, strong mechanical properties, high chemical/electrochemical stability, and superior ionic/electronic conductivity. For example, Kwon et al. reported a multifunctional binder incorporating Meldrum’s acid *via* a systematic molecular level design for Si anodes, namely, 5-methyl-5-(4-vinlybenzyl) Meldrum’s acid (K) as a ketene precursor for cross-linking and initial covalent attachment to Si, styrene (S) for stiffness, methyl methacrylate (M) for flexibility, and finally lithium 2-methyl-2-(4-vinylbenzyl) malonate (C) for the self-healing effect (Kwon et al., 2014). They demonstrated that the self-healing effect results in significant enhancement by a modular approach based on the monomer design and varied monomer ratio, which can also be regarded as a direct guideline on how to design binders for next-generation Si-based anodes.

## Conclusion and Perspective

Intrinsic volume expansions and low ionic/electronic conductivity hinder the commercialization of Si-based anode materials for high energy density LIBs. Binders play a key role in maintaining internal electrical contact, high capacity, and long-term stability for Si-based anodes in LIBs despite a small percentage of quality. By now, the binder content of an anode is generally ∼10% in academia, which is higher than that of commercial Si anodes (∼5%). Binders have no direct contribution to capacity; thus, reducing the content of the binder while ensuring its functionality is conducive to commercialization. In this work, we summarized the polymer structures (especially the hydrogen bonds, covalent bonds, and functional groups) of various linear binders and their advantages and disadvantages in Si anodes. Based on the literature review, the reconstruction of the linear binders into hybrid binders, especially the three-dimensional cross-linked binder, can provide more attachment sites and active space between binders and Si particles, which is currently the most promising way to design a feasible binder for commercial Si anodes. More research studies are needed to be carried out to further understand the working and failure mechanisms of binders for Si-based anodes for LIBs *via in situ* characterization techniques and systematic design principles, such as cryoelectron microscopy (Huang et al., 2019), *in situ* IR and finite element simulations (Hu et al., 2021), secondary ion mass spectrometry (ToF-SIMS) (Pereira-Nabais et al., 2014), DFT calculations (Lee et al., 2015), and full-cell matching with Ni-rich cathodes (Kim et al., 2021).
